# Un abcès du psoas compliquant une appendicite aiguë

**DOI:** 10.11604/pamj.2015.22.231.8201

**Published:** 2015-11-11

**Authors:** Ammar Mahmoudi, Mabrouk Abdelali

**Affiliations:** 1 Service de Chirurgie Générale et Digestive, CHU Fattouma Bourguiba de Monastir, Monastir, Tunisie; 2 Service d’ Imagerie Médicale, CHU Fattouma Bourguiba de Monastir, Monastir Tunisie

**Keywords:** Appendicite aiguë, abcès du psoas, scanner, antibiothérapie, chirurgie, acute appendicitis, psoas abscess, CT scan, antibiotherapy, Surgery

## Image en medicine

Primitif ou secondaire, l'abcès du psoas est une entité rare. Son diagnostic étiologique peut s'avérer délicat. En ce sens l'apport de l'imagerie et en particulier de la tomodensitométrie est fondamental. Malgré les examens d'imagerie, l'origine de l'abcès peut demeurer inconnue et découverte qu'en peropératoire (66% des cas). L'appendicite occupe la deuxième place (16%) après la maladie de Crohn (60%) parmi les étiologies digestives des abcès du psoas. L'appendice siège dans 26 à 65% des cas en position rétro-cæcale. Dans cette position, l'appendice peut loger dans une fossette péritonéale ou en rétropéritonéale et l'infection évoluer vers la formation d'un abcès, la diffusion vers l'espace rétropéritonéal, et rarement se compliquer d'un abcès du psoas. L'appendicite aiguë rétro-cæcale réalise un tableau clinique insidieux, atypique dans plus de la moitié des cas. A ce propos nous en rapportons une observation d'un homme de 34 ans, diabétique insulinodépendant, ayant présenté une douleur de la fosse iliaque et lombaire droite depuis une semaine. A l'examen, il était fébrile à 38,5°C et il y avait un psoïtis, une sensibilité de l'hémi-abdomen droit, et une masse para-ombilicale et lombaire droite. Il existait une hyperleucocytose à 24000/mm^3^ et une CRP à 109 mg/l. La tomodensitométrie abdominale (A, B, C, D) avait montré un abcès du psoas droit d'origine appendiculaire. Par une incision de Mac Burney, l'appendice était rétro-cæcal abcédé compliqué d'un abcès du psoas. Il a été réalisé une appendicectomie, une évacuation de la collection purulente du psoas et un drainage. Sous antibiothérapie, les suites opératoires étaient simples.

**Figure 1:**
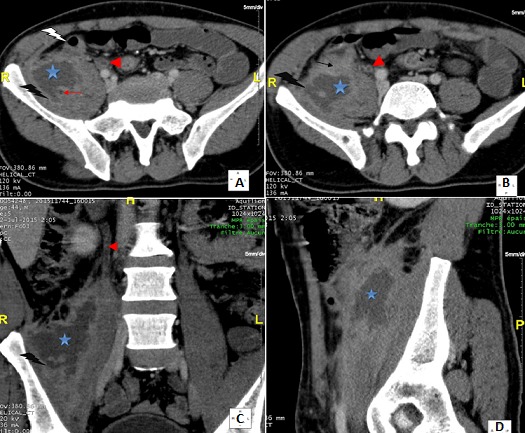
A): coupe tomodensitométrique axiale passant par la fosse iliaque droite réalisée après injection intraveineuse de produit de contraste (temps portal): collection liquidienne intéressant le muscle psoas droit de contours irréguliers avec une coque périphérique épaisse qui se rehausse après injection de PDC. Absence de visualisation de l'appendice en latéro-caecal interne. Absence d’épaississement de la dernière anse iléale. Présence d'une petite image à centre hypodense au sein de cette collection: bout d'appendice. A noter un rehaussement pariétal circonférentiel de l'uretère au contact (urétérite réactionnelle); B): coupe tomodensitométrique axiale passant par la fosse iliaque droite réalisée après injection intraveineuse de produit de contraste (temps portal): Collection liquidienne intéressant le muscle psoas droit de contours irréguliers avec une coque périphérique épaisse qui se rehausse après injection de PDC. Absence de visualisation de l'appendice en latéro-cæcal interne. Noter la disparition du liseré graisseux de séparation entre le psoas et le bas fond cæcal avec densification de la graisse tout autour. A noter un rehaussement pariétal circonférentiel de l'uretère au contact (urétérite réactionnelle); C): coupe tomodensitométrique coronale passant par la fosse iliaque droite réalisée après injection intraveineuse de produit de contraste (temps portal): Noter les contours irréguliers de la collection intramusculaire et sa coque périphérique épaisse qui se rehausse après injection de PDC Noter l'urétérite réactionnelle associée; D): coupe tomodensitométrique sagittale passant par le muscle ilio-psoas droit réalisée après injection intraveineuse de produit de contraste (temps portal): l’épicentre de la collection siège au niveau de la portion rétro-cæcale du muscle psoas

